# Polyamines and Epithelial Barrier Loss During HIV Infection

**DOI:** 10.3390/pathogens15101069

**Published:** 2026-10-09

**Authors:** Shanmuga S. Mahalingam, Cheryl M. Cameron, Brian Richardson, Mark J. Cameron, Andre da Silva Paes, Andres Pinto, Banumathi Tamilselvan, Sangeetha Jayaraman, Jeffrey M. Jacobson, George Yendewa, Pushpa Pandiyan

**Affiliations:** 1 Department of Biological Sciences, School of Dental Medicine, Case Western Reserve University, Cleveland, OH 44106, USA; 2 Systems Biology and Bioinformatics Program, Department of Nutrition, School of Medicine, Case Western Reserve University, Cleveland, OH 44106, USA; 3 Department of Periodontics, School of Dental Medicine, Case Western Reserve University, Cleveland, OH 44106, USA; 4 Department of Oral Medicine, School of Dental Medicine, Case Western Reserve University, Cleveland, OH 44106, USA; 5 Case AIDS Clinical Trials Unit, Department of Medicine, School of Medicine, Case Western Reserve University, Cleveland, OH 44106, USA; 6 Division of Infectious Diseases and HIV Medicine, University Hospitals Cleveland Medical Center, Cleveland, OH 44106, USA; 7 Department of Pathology, School of Medicine, Case Western Reserve University, Cleveland, OH 44106, USA; 8 Department of Molecular Biology and Microbiology, School of Medicine, Case Western Reserve University, Cleveland, OH 44106, USA; 9 Center for AIDS Research, Case Western Reserve University, Cleveland, OH 44106, USA

**Keywords:** polyamines, epithelial barrier, immune dysfunction, people living with HIV (PWH), microbiome

## Abstract

Despite effective antiretroviral therapy (ART), people with HIV (PWH) continue to experience chronic oral mucosal disease characterized by persistent T-cell inflammation, regulatory T cell (Treg) dysfunction, impaired CD8^+^ tissue resident cell (TRM) immunity and microbial dysbiosis. Emerging evidence implicates dysregulated polyamine metabolism and mucosal T-cell dysfunction as key mediators of these pathological processes. However, the effects of polyamines on the oral mucosal epithelium remain largely unexplored. The paradoxical dose-dependent effects of polyamines on oral epithelial barrier integrity are critically discussed, and a unified mechanistic model is proposed. This paper also highlights single-cell transcriptomic data from our laboratory that implicates epithelial cell barrier dysfunction in PWH and offers a perspective on how it may worsen persistent inflammation even after combined anti-retroviral treatment. This hypothesis reports current knowledge on how HIV-induced polyamine overload and *Fusobacterium*-derived putrescine could drive oral epithelial barrier disruption through interconnected mechanisms involving oxidative stress, α6β4 integrin–laminin-332 signaling, HIF-1α stabilization, tight junction disorganization, and innate immune dysregulation. Finally, we outline key future research directions, including therapeutic targeting of polyamine oxidation to restore oral mucosal homeostasis in PWH.

## 1. Introduction

Despite effective ART, PWH continue to experience chronic oral mucosal disease characterized by epithelial inflammation, barrier dysfunction, microbial dysbiosis, and impaired mucosal immunity. These abnormalities may increase susceptibility to infection, promote systemic inflammation, and impair quality of life. However, the mechanisms sustaining oral immune dysfunction remain poorly understood, and mechanism-based therapies to restore barrier integrity are lacking. Our studies identify polyamine metabolism as a novel regulator of T cell dysregulation and oral mucosal immunity during HIV infection [1,2,3,4,5]. Integrated proteomic, transcriptomic, metabolomic, and human tonsil organoid studies demonstrate increased polyamine synthesis, elevated salivary putrescine, dysregulated Treg/Th17 responses, impaired CD8^+^ TRM responses, and oral microbial dysbiosis in PWH [2,6,7,8,9,10,11]. Importantly, inhibiting polyamine synthesis restored T cell functions, supporting this pathway as a potential therapeutic target [2,12,13,14]. Dysregulation of polyamine metabolism is increasingly recognized as both a consequence of and contributor to chronic inflammation and mucosal injury [4,5,7,15,16,17,18]. Emerging evidence links polyamine metabolism with oxidative stress, HIF-1α signaling, epithelial metabolism, and barrier integrity [16,19,20,21,22], but this axis has not been systematically examined in excessive polyamine contexts and HIV-associated oral disease. Whether dysregulated polyamines also directly compromise epithelial barrier integrity, which may cause dysbiosis and “leaky oral mucosa”, remains unknown. This perspective article highlights the evidence linking excessive polyamine accumulation to epithelial barrier dysfunction and mucosal immune dysregulation and proposes a unified framework for understanding and targeting this pathway in PWH.

### 1.1. Keratinocytes as the Primary Barrier Epithelial Cells

The oral epithelial barrier, formed predominantly by keratinocytes, integrates structural, adhesive, and immune mechanisms to protect the mucosa from microbial and environmental insults [23]. Barrier integrity is maintained by tight junction proteins such as occludin and claudin (OCLN, TJP1/ZO-1, CLDN1, CLDN4, CLDN7) that restrict paracellular permeability, E-cadherin (CDH1) and desmosomes that maintain cell–cell cohesion, and keratin networks and α6β4 integrin–laminin interactions that provide mechanical stability and basement-membrane attachment [24,25,26,27,28,29,30,31]. Keratinocytes additionally function as innate immune sentinels through TLRs and NOD-like receptors, responding to microbial signals by producing IL-1β, IL-6, TNF, CXCL8, CCL20, CXCL10, and antimicrobial peptides such as β-defensins and S100 proteins [32,33,34,35,36]. These coordinated mechanisms vary with epithelial differentiation and keratinization, with non-keratinized mucosa relying particularly on junctional integrity and epithelial turnover, and may have bi-directional cross-talk with T cells [37]. Thus, the oral epithelium functions as a dynamic barrier that integrates cell–cell adhesion, extracellular-matrix signaling, microbial sensing, and immune responses, supported by underlying stromal and vascular compartments.

### 1.2. α6β4 Integrin Signaling in Oral Epithelial Barrier Homeostasis

The α6β4 integrin (ITGA6/ITGB4) is a major signaling receptor in stratified epithelia, including the oral mucosa, whose functional importance extends far beyond simple anchorage to the basement membrane [31,38]. Rather than serving merely as a structural tether, α6β4 operates as a multifunctional signaling hub that coordinates epithelial survival, migration, proliferation, and responses to tissue injury [30]. Understanding its diverse roles is essential for appreciating how chronic epithelial stress during HIV infection may translate into persistent barrier dysfunction. At the most fundamental level, α6β4 integrin maintains epithelial adhesion and structural integrity by binding primarily to laminin-332 within the basement membrane [39]. As a key component of hemidesmosomes, it physically links epithelial cells to the extracellular matrix and to the intermediate filament cytoskeleton, thereby providing the mechanical stability necessary to sustain epithelial barrier integrity under the constant physical and microbial stresses encountered at mucosal surfaces. Beyond this structural role, α6β4 actively promotes epithelial cell survival by also engaging the PI3K–AKT signaling axis, a function that becomes particularly critical during tissue stress or injury when epithelial cells must resist apoptosis to maintain barrier continuity [40,41].

The receptor’s role in wound healing reveals an additional layer of functional complexity. Following epithelial injury, α6β4 undergoes dynamic changes in its hemidesmosomal associations and transitions into a pro-migratory signaling configuration, engaging downstream pathways including FAK/Src, PI3K–AKT, and small GTPases to promote epithelial cell migration and proliferation during wound repair [40,41,42]. This capacity to switch from a primarily adhesion receptor to a pro-migratory signaling receptor makes α6β4 a central coordinator of the epithelial injury response. The receptor further amplifies growth factor signaling by cooperating with receptor tyrosine kinases, particularly EGFR [41]. This cooperation is important for epithelial proliferation and regeneration following mucosal injury. Finally, α6β4 signaling influences NF-κB and other inflammatory pathways, thereby affecting epithelial production of cytokines and chemokines and creating an important mechanistic connection between epithelial adhesion, tissue injury, and immune responses [43].

α6β4 integrin is also an important component in the epithelial sensing and response system and could be especially relevant in the context of chronic infection, microbiome dysbiosis, and aging, where persistent microbial or inflammatory stress could progressively alter α6β4 signaling and impair epithelial repair mechanisms. The consequence of such dysregulation is a self-reinforcing cycle in which barrier disruption drives inflammation, inflammation impairs repair, and impaired repair leads to further mucosal damage, a cycle that may be particularly difficult to interrupt in the setting of chronic HIV infection. Laminin-332, the primary ligand for α6β4 integrin, is assembled from three genetically distinct subunits encoded by *LAMA3*, *LAMB3*, and *LAMC2* [44]. These proteins together form a functional heterotrimer, which then binds to the α6β4 integrin receptor that is composed of *ITGA6* and *ITGB4* subunits to anchor epithelial cells to the basement membrane. Among these subunits, LAMC2 (laminin γ2) deserves particular attention in the context of this review. Beyond its structural role in laminin-332 assembly, LAMC2 is implicated in epithelial injury responses, cell migration, cancer invasion, and hypoxia-related biology [45], making it a potentially informative marker of epithelial stress in the HIV-infected oral mucosa. There is direct experimental evidence that α6β4 integrin signaling can activate HIF-1α, although the strongest evidence currently derives from cancer epithelial models rather than normal oral epithelium [46,47]. HIF-1α is also known to impact defensin expression, mucin production and adherens junctions in intestinal epithelial cells, with direct implications for barrier maintenance [48,49,50,51,52]. At the onset of arthritis, HIF-1α is also one of the most upregulated genes in these cells and was shown to function as a transcriptional repressor of RIPK3, a central mediator of necroptotic cell death [53]. HIF-1α binds hypoxia response elements (HREs) within the RIPK3 promoter. By suppressing RIPK3, HIF-1α shifts the balance of cell death away from pathological necroptosis and toward physiological apoptosis, thereby preserving barrier integrity. While HIF-1α consistently acts as a barrier-protective factor in intestinal epithelial cells, its role in airway epithelium is more nuanced, simultaneously facilitating pathogen uptake while activating innate immune defenses, a duality that may well extend to the oral mucosal environment. Taken together, intestinal and airway epithelial models show that HIF-1α is a pleiotropic regulator of epithelial barrier function, maintaining metabolic homeostasis under inflammatory stress and modulating innate immune responses. These findings collectively highlight the importance of investigating HIF-1α regulation directly within oral epithelial cells, rather than relying solely on extrapolations from intestinal or airway models. The relationship between α6β4 and HIF-1α involves HIF-1α stabilization and nuclear accumulation rather than transcriptional upregulation of HIF1A, a distinction with important implications for understanding how this pathway operates under hypoxic and normoxic conditions. Specifically, we propose that during chronic HIV infection, the relevant stressors include persistent microbial dysbiosis, immune-mediated epithelial injury, and epithelial remodeling that progressively compromises barrier integrity, generating further microbial exposure and perpetuating mucosal inflammation. The α6β4–HIF-1α axis represents both a mechanistic explanation for persistent epithelial dysfunction in PWH and a potentially tractable therapeutic target. Studying this axis requires a comprehensive investigative approach in oral epithelial cells under chronic hyperinflammatory states relevant to HIV infection.

### 1.3. Polyamine Metabolism and Epithelial Barrier Regulation

Polyamines such as putrescine, spermidine, and spermine are ubiquitous polycationic molecules essential for immune cell growth, proliferation, and differentiation [2,4,7,18,54]. Their intracellular concentrations are tightly regulated through a balance of biosynthetic and catabolic pathways [2,54]. Polyamines can suppress excessive inflammatory signaling in some settings while supporting appropriate epithelial antimicrobial functions in others, reflecting the complex dose- and context-dependent biology of this metabolite class. On the biosynthetic side, ODC1 (ornithine decarboxylase) serves as the rate-limiting enzyme for putrescine synthesis. On the catabolic side, Spermidine/Spermine N^1^-Acetyltransferase 1 (SAT1), Spermine Oxidase (SMOX), and peroxisomal N^1^-acetyl-spermine/spermidine oxidase (PAOX) catalyze the back-conversion and oxidative degradation of higher polyamines, generating reactive oxygen species (ROS) as obligate byproducts of these reactions [55]. The relationship between polyamines and epithelial tight junction proteins shows protective roles in the context of normal tissue homeostasis [56]. Polyamine depletion with DFMO reduces occludin protein without substantially reducing OCLN mRNA, firmly establishing that polyamines upregulate occludin through post-transcriptional mechanisms governing translation and protein stability [57]. The specific pathway involves CHK2-dependent phosphorylation of the RNA-binding protein HuR, which in its phosphorylated state binds to OCLN mRNA and promotes its translation [58]. In differentiated intestinal epithelial cells, the functional consequences of polyamine depletion are substantial, encompassing reductions in occludin, ZO-1/TJP1, ZO-2, claudin-2, and claudin-3, accompanied by decreased transepithelial electrical resistance (TEER) and increased paracellular permeability [15]. The reversibility of these changes upon polyamine repletion confirms that polyamine availability is a direct determinant of tight junction protein expression and barrier function. Strong mechanistic evidence also links polyamine availability to E-cadherin (CDH1) where polyamine depletion decreases c-MYC expression, which in turn reduces CDH1 promoter activity and CDH1 mRNA levels [59]. Therefore, this mechanistic linkage between adherens-junction and tight junction integrity means that loss of polyamines can disrupt the epithelial barrier. The disruption may involve at least two parallel and potentially synergistic pathways: direct suppression of tight junction protein translation and indirect destabilization of tight junctions through adherens-junction loss. Beyond their roles in regulating junctional proteins, polyamines also modulate the innate immune sensing capacity of epithelial cells. Polyamine depletion has been shown to decrease TLR2 expression, contributing to increased epithelial permeability and potentially impairing the ability of epithelial cells to appropriately sense and respond to microbial stimuli [60]. This creates a potentially important feedback loop in which polyamine dysregulation simultaneously compromises the physical barrier and impairs the innate mucosal immune responses.

However, a critical paradox in the polyamine–barrier literature must be carefully considered and clearly articulated, as it has direct implications for the mechanistic hypotheses proposed in the review in the context of chronic infections such as HIV. It is important to note that the direction of polyamine effects on innate immune signaling is context-dependent [61,62,63]. The capacity for reactive oxygen species (ROS) generation during polyamine catabolism is central to understanding how excessive polyamines and dysregulated polyamine metabolism can drive oxidative stress-mediated epithelial injury [16,45]. The evidence that excess putrescine can directly disrupt tight junctions comes from a landmark high-throughput screen for regulators of intestinal epithelial barrier function [64]. In this study, putrescine was identified as a tight-junction disruptor in an imaging-based screen using CaCo-2 and T84 cells, and its barrier-disruptive effects were subsequently confirmed in ex vivo mouse colon tissue and in vivo mouse models. Administration of putrescine to mice caused increased intestinal permeability, reduced transepithelial electrical resistance (TEER), increased inflammatory signaling, increased microbial translocation-associated pattern-recognition receptor ligands in mesenteric lymph nodes, exacerbation of colitis, increased intestinal shedding of viable *Citrobacter rodentium*, and elevated inflammatory cytokines in colon tissues. Mechanistically, colonic epithelial cells from putrescine-treated mice showed increased expression of genes regulating metal binding, oxidative stress, and cytoskeletal organization and contractility, providing a plausible basis for tight-junction disruption. Importantly, co-administration of taurine with putrescine blocked tight-junction disruption and the exacerbated inflammation, suggesting that the barrier-disruptive effects of putrescine are pharmacologically reversible.

A particularly compelling mechanistic explanation for the barrier-disruptive effects of excess putrescine centers on its oxidative catabolism by SMOX and PAOX, which generates hydrogen peroxide and aldehyde byproducts as obligate products of the reaction [7,54,55,65]. This oxidative catabolism could generate a pathogenic cascade that mechanistically links elevated putrescine to tight-junction disorganization through oxidative stress and cytoskeletal remodeling. This model is mechanistically compelling, because it explains both the rapidity of putrescine-induced barrier disruption and the observation that putrescine-treated epithelial cells show altered cytoskeletal gene expression [64]. The finding that taurine, an antioxidant, can block putrescine-induced barrier disruption is consistent with this oxidative stress-centered mechanism and suggests that pharmacological antioxidant strategies could potentially rescue barrier function in settings of polyamine excess. We propose that under conditions of normal polyamine availability, polyamines promote CHK2-dependent HuR phosphorylation and occludin translation. Under conditions of polyamine excess, the dominant effect may shift to SMOX/PAOX-mediated ROS generation and cytoskeletal disruption, producing tight-junction disorganization even when total occludin protein may not be substantially reduced. These two mechanisms, i.e., translational regulation of occludin and cytoskeletal disruption of tight-junction organization, are therefore not mutually exclusive and may operate in parallel depending on the magnitude and duration of polyamine dysregulation. Overall, both polyamine excess and polyamine depletion can compromise epithelial barrier integrity, although they act through distinct and mechanistically separable pathways. In the setting of HIV infection, dysregulated polyamine metabolism may impair HIF-1α activity in oral epithelial cells, thereby weakening barrier integrity through disrupted junctional maintenance, or increased susceptibility to necroptosis and impaired epithelial tissue repair. A direct physical interaction between the polyamine catabolic enzyme SAT1 and HIF-1α, showing that a polyamine catabolism mediator can downmodulate HIF-1α function [20], supports this possibility. However, it remains to be seen whether exogenous putrescine or polyamine oxidation directly reduces HIF-1α protein or HIF1A mRNA in epithelial cells. This is particularly relevant to HIV-associated oral disease, as any HIV-driven reduction in HIF-1α activity could upregulate RIPK3, promoting necroptotic cell death and accelerating barrier disruption. Because most existing studies have examined epithelial cells in non-oral systems, these proposed mechanisms must be experimentally validated in oral mucosal models.

### 1.4. Does the Loss of Epithelial Barrier Functions Co-Ordinate Dysbiosis and T Cell Immunometabolic Dysfunction or Vice Versa

**Figure 1:**
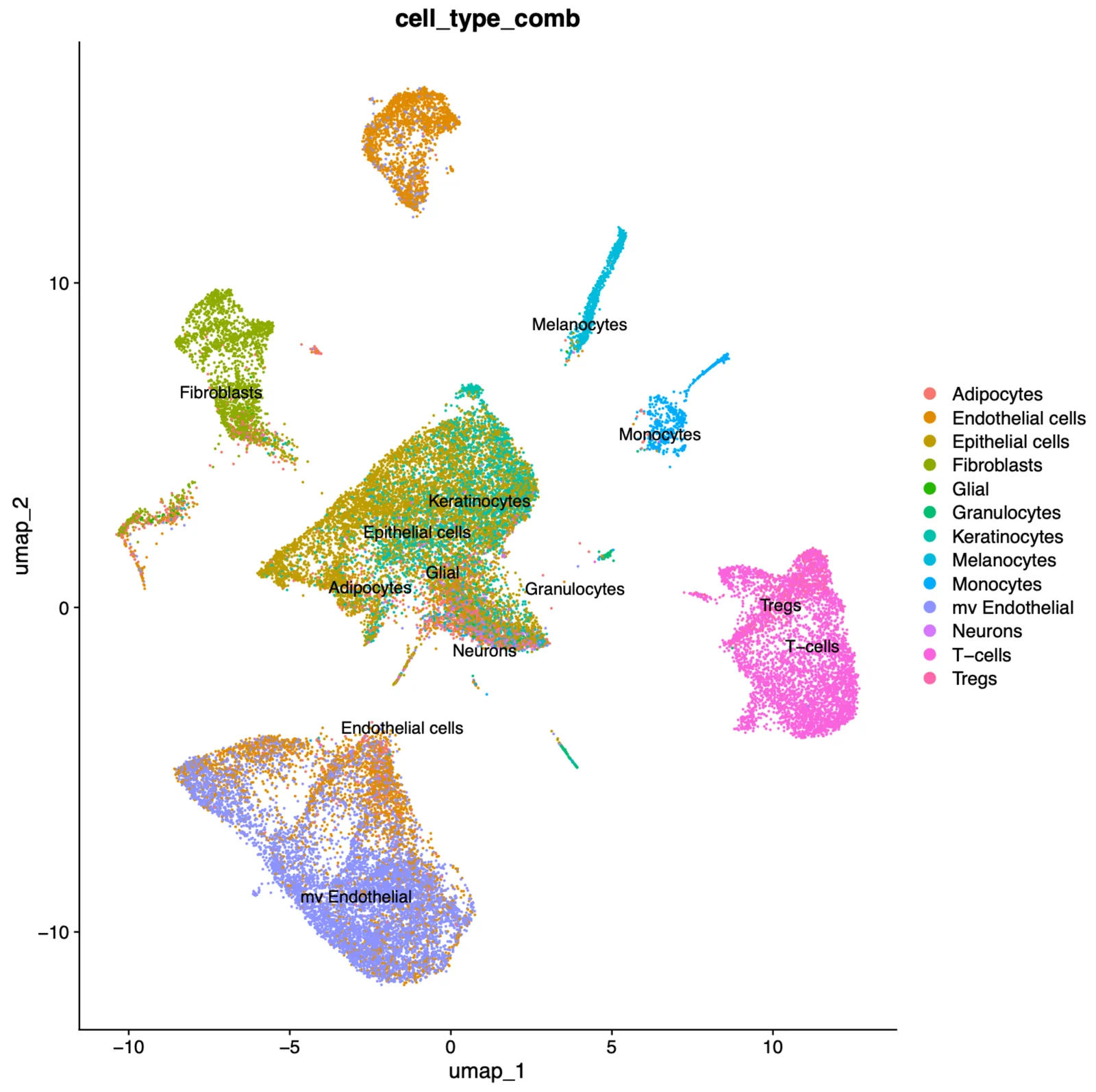
Single-cell landscape of gingival tissue. Uniform manifold approximation and projection (UMAP) of single cells from thirteen gingival donors, uninfected control (n = 3) and PWH (n = 10) shown together. Cell identities were assigned with SingleR using the Blueprint and ENCODE reference and grouped into thirteen populations for display. Colors denote cell type as indicated. Epithelial cells are analyzed subsequently.

**Figure 2:**
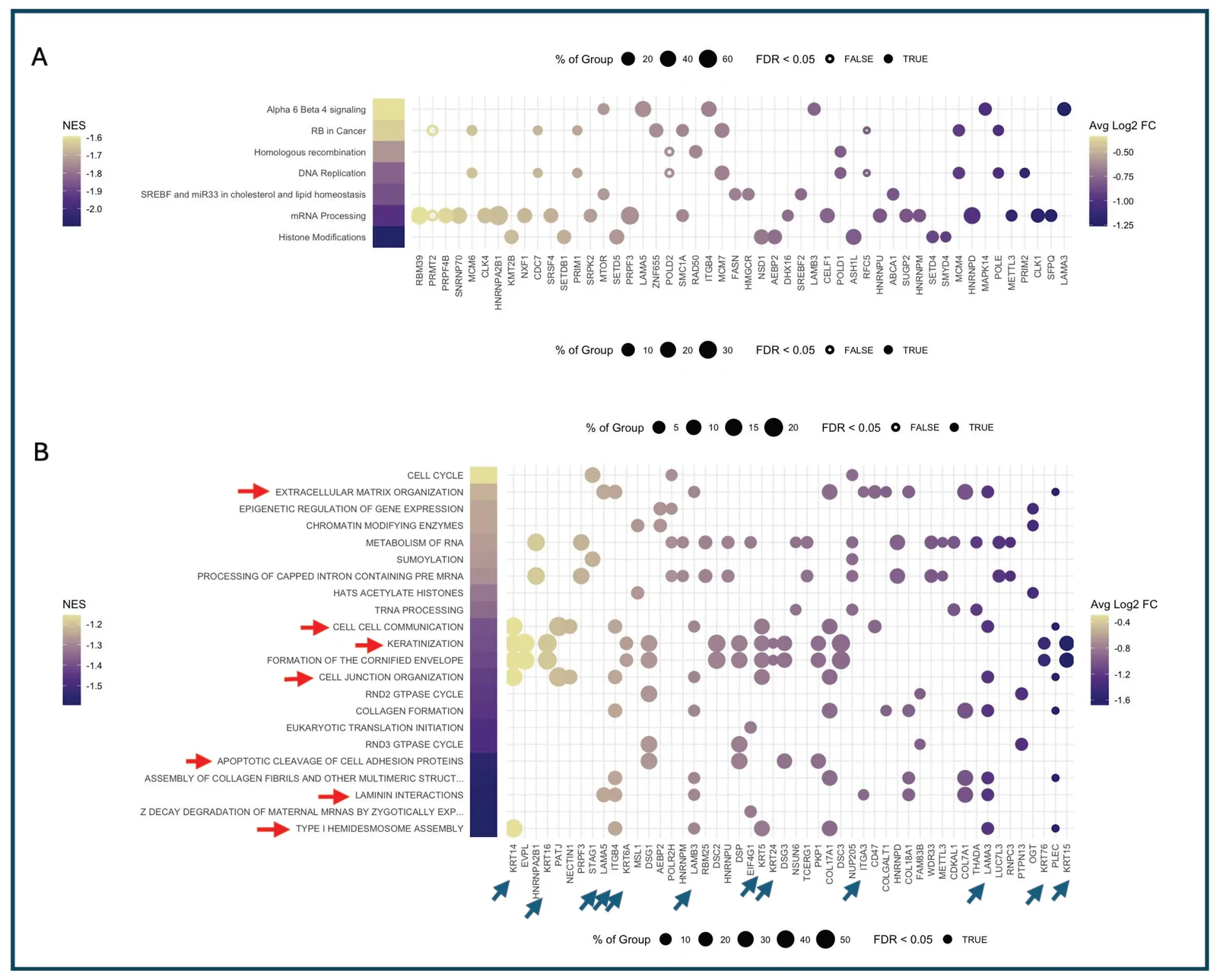
Reduced α6β4 signaling, keratinization and cell junction programs in gingival epithelial cells, PWH versus control group. Gene set enrichment analysis (WikiPathways, ConsensusPathDB) (**A**) and (Reactome, MSigDB) (**B**) of epithelial cells from gingival tissue on genes ranked by signed log *p* value, PWH (n = 10) with uninfected control (n = 3). Pathways were selected and ranked on nominal *p* value and the top 25 are shown, with positive normalized enrichment scores (NES) in the upper panel and negative NES in the lower panel. The color bar to the left of each panel shows the NES. Each dot is a leading edge gene within that pathway, sized by the percentage of the gene set represented and colored by the average log2 fold change between groups. Filled dots indicate genes with a false discovery rate < 0.05, open dots ≥ 0.05. Red arrows mark the key keratinization and cell junction organization pathways relevant to our hypothesis. Blue arrows mark the leading edge genes KRT14, KRT16, ITGB4, LAMB3, KRT5 and LAMA3.

In the context of chronic HIV infection, mucosal immune dysregulation is compounded by oral microbial dysbiosis that creates a self-amplifying cycle of polyamine overproduction and immune dysfunction [2,4,5,8,9,17,18]. Elevated *Fusobacteria* abundance in the oral microbiome positively correlates with salivary polyamine levels in PLWH, identifying *Fusobacterium nucleatum* (FN) as a substantial source of immunomodulatory polyamines in the HIV-infected oral mucosa [2,4]. The additive effects of FN and HIV on T cell dysfunction are significant: FN-derived polyamines compound the immunosuppressive effects of HIV-induced polyamine synthesis, producing a degree of T cell dysfunction in the oral mucosa of PLWH that exceeds what either insult produces independently [4]. This synergistic immunosuppression has important implications for mucosal defense, as it suggests that therapeutic strategies targeting polyamine metabolism must account for both host and microbial sources of polyamine production. Polyamines play a critical mechanistic role in suppressing TRM cell function through the process of EIF5A hypusination, in which spermidine serves as the obligate substrate for the post-translational modification of the translation factor EIF5A [4]. Hypusinated EIF5A is required for the efficient translation of a subset of mRNAs, and polyamine-driven EIF5A hypusination in the HIV-infected oral mucosa reduces expression of IFN-γ and key TRM signature markers. The functional consequences of this suppression are amplified by a feed-forward mechanism: IFN-γ itself promotes TRM cell induction and proliferation, such that polyamine-dependent repression of IFN-γ production impairs the very signals required for maintaining the TRM compartment [2,4]. This creates a self-reinforcing deficit in mucosal tissue-resident immunity that may be particularly difficult to reverse in the setting of chronic HIV infection. This led us to propose that HIV-dependent polyamine dysregulation, TRM defects, and *Fusobacterium*-derived putrescine contribute to epithelial barrier loss perpetuating immune dysregulation. Our initial examination of gingival tissues from PWH supports this tenet. Single-cell RNA sequencing of gingival tissue from thirteen donors, comprising three donors in the uninfected group and ten donors in the PWH group, captured a diverse cellular landscape within the gingiva. We integrated the single-cell transcriptomes from all donors and used UMAP (Seurat library; version 5.2.0) to visualize the transcriptionally distinct cellular populations. Cell identities were then assigned based on their gene-expression profiles using SingleR with the Blueprint and ENCODE reference datasets. This analysis resolved the gingival tissue into thirteen distinct cell populations, representing the major cellular compartments present in the tissue (Figure 1). We subsequently identified and isolated the epithelial cell compartment for further analysis, enabling us to resolve the epithelial populations in greater detail and characterize their distinct molecular features. To further characterize the molecular differences within the gingival epithelial compartment, we performed gene set enrichment analysis comparing epithelial cells from people living with HIV (PLWH; n = 10) with those from uninfected controls (n = 3). Genes were ranked according to their signed log *p* value, and pathway enrichment was evaluated using complementary pathway resources indicated. The analyses identified pathways that were positively or negatively enriched between the two groups, with the top 25 pathways ranked according to nominal *p* value displayed in the respective panels. Among the enriched pathways, keratinization and cell junction organization were particularly notable and are highlighted by circles (Figure 2). Taken together, our previously published findings demonstrating polyamine dysregulation in gingival tissues of PWH [4,5,7,8,9,17] together with evidence of impaired epithelial barrier integrity, suggest a strong bidirectional relationship between these phenomena in PWH. However, the single-cell RNA sequencing results presented here should be interpreted with caution and validated through careful assessment of protein expression and functional activity of key components in the identified pathways. Whether dysbiosis and increased oral mucosal permeability result from epithelial barrier disruption and, in turn, modulate T-cell responses in PWH remains to be determined in the future studies.

Another recent work has revealed an additional and mechanistically distinct pathway through which HIV-associated CD8^+^ T cell dysfunction drives epithelial barrier disruption. PPARγ downregulation in colonic CD8^+^ T cells from PWH on ART disrupts lipid metabolism in these cells and promotes contact-dependent epithelial apoptosis, leading to intestinal barrier dysfunction that persists despite viral suppression [66]. Pharmacological activation of PPARγ restored metabolic fitness in CD8^+^ T cells and reduced epithelial damage, revealing a potential therapeutic target for mucosal repair in PWH. This finding is significant for the present review because it demonstrates that T cell metabolic reprogramming, operating through a mechanism entirely distinct from polyamine metabolism, can independently drive epithelial barrier failure during HIV infection. The convergence of polyamine-mediated immunosuppression and PPARγ-dependent CD8^+^ T cell dysfunction on the common endpoint of epithelial barrier disruption underscores the multifactorial nature of HIV-associated mucosal pathology and suggests that effective therapeutic restoration of barrier integrity may require addressing multiple parallel mechanisms simultaneously. Beyond their effects on CD8^+^TRM cells, polyamines also contribute to regulatory T cell (Treg) dysregulation in the HIV-infected oral mucosa, suggesting that chronic infection and dysbiosis drive a coordinated immunometabolic dysfunction that simultaneously impairs effector immunity and dysregulates mucosal immune homeostasis [3,4,5,17,18,67]. Thus, TRM cell attrition, Treg dysfunction, and antigen-independent immune hyperactivation may collectively contribute to epithelial barrier dysfunction by compromising the immune support required for epithelial maintenance and repair during chronic HIV infection [4]. It is also tempting to speculate that these mechanisms establish a paracrine feedback loop in which epithelial barrier integrity and immune mechanisms that normally maintain resident microbiota are simultaneously impaired. This reciprocal dysfunction may thereby sustain mucosal inflammation, dysbiosis, and progressive barrier disruption during chronic HIV infection.

## 2. Conclusions

**Figure 3:**
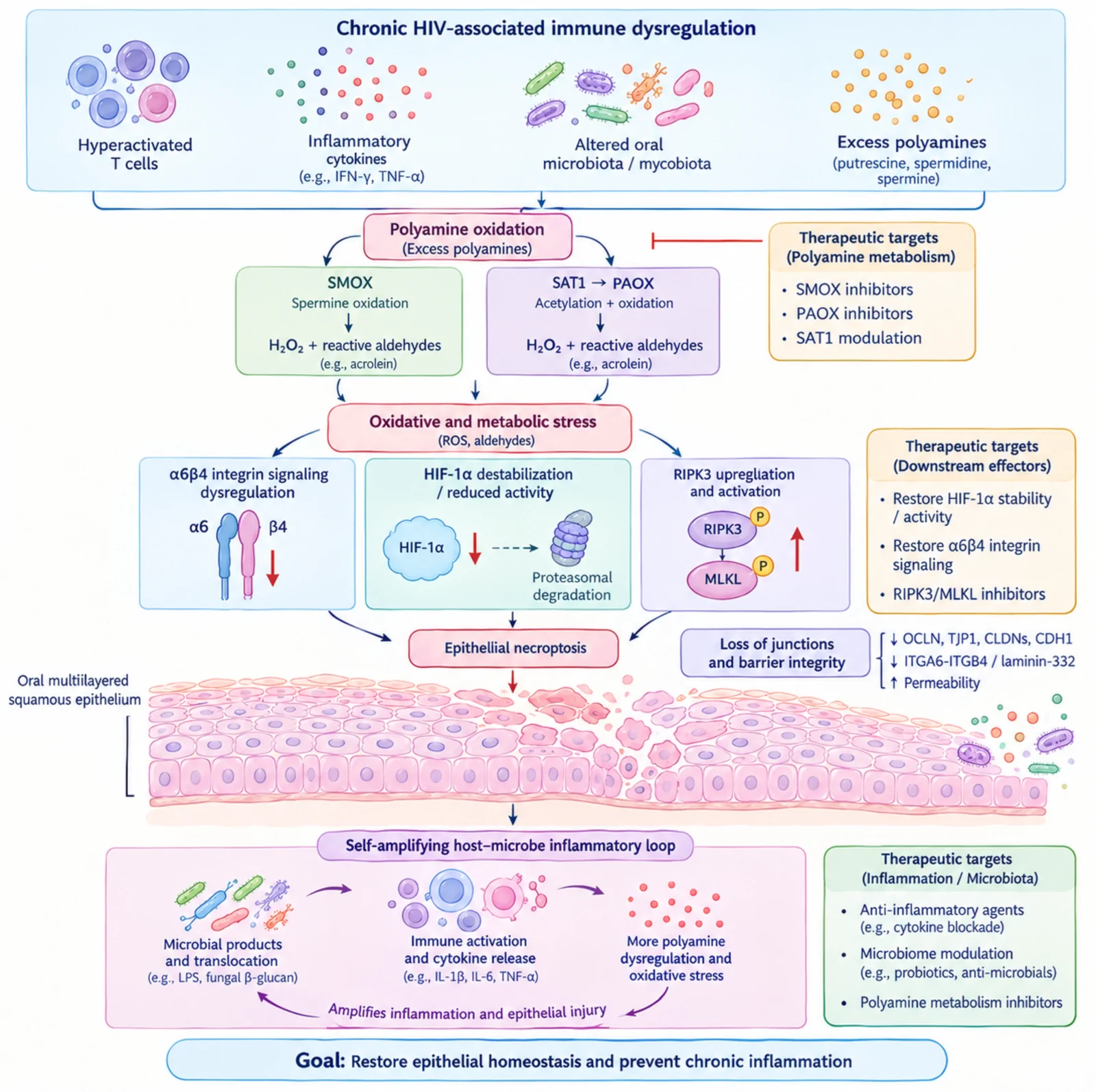
Unified mechanistic model in which HIV-induced polyamine dysregulation may drive oral epithelial barrier dysfunction. (The cartoon was created by Dr. Pandiyan using an Artificial Intelligence tool).

This review proposes a unified mechanistic model in which HIV-induced polyamine dysregulation may drive oral epithelial barrier dysfunction through three interconnected pathways (Figure 3). The first disrupts junctional integrity by impairing CHK2/HuR-dependent occludin translation, suppressing ZO-1/TJP1, and reducing E-cadherin via c-MYC-dependent transcriptional suppression. The second compromises innate immune sensing by reducing TLR2 expression and altering antimicrobial peptide and cytokine production, creating conditions favorable for dysbiosis-driven polyamine dysregulation and barrier disruption. The third drives metabolic reprogramming through SMOX/PAOX-mediated ROS generation, disrupting α6β4 integrin-cytoskeletal organization and tight-junction integrity (ZO-1/TJP1) while potentially suppressing HIF-1α-dependent barrier-protective and anti-necroptotic programs. We consider the third mechanism the most plausible hypothesis, especially in the context of HIV-chronic inflammation based on our data presented here and previous findings, which will be investigated in future studies. These pathways converge on barrier dysfunction and permit microbial translocation, perpetuating a self-reinforcing cycle of mucosal inflammation. One limitation of the proposed hypotheses and mechanisms in this article is that they have not been directly demonstrated in the oral mucosa but are inferred from studies in other biological systems. Therefore, these mechanisms should be interpreted with caution until validated mechanically and functionally in oral epithelial cells, which will be a major focus of future studies. Critically, the relationship between polyamines and barrier integrity is dose-, compartment-, and context-dependent. While physiological polyamine levels maintain barrier function, excess putrescine, particularly through oxidative catabolism, may disrupt tight junctions and may suppress HIF-1α-dependent repair programs, a paradox that must be explicitly addressed in future experimental designs. Experimentally, this complexity necessitates comprehensive measurement of TLR signals alongside integrins, antimicrobial peptides, and inflammatory mediators. Several key questions remain unresolved, including whether the α6β4–HIF-1α axis operates differentially across keratinized and non-keratinized mucosal compartments, the direct effects of putrescine on HIF-1α specifically in oral epithelial cells, the functional properties of oral mucosal Tregs and TRM cells in modulating barrier functions during chronic HIV infection, and whether restoring dysbiosis normalizes polyamine dysregulation in virologically suppressed individuals. Collectively, the evidence positions polyamine-driven reprogramming of junctional integrity, microbial sensing, and epithelial metabolism as a tractable and mechanistically coherent target for therapeutic intervention in HIV-associated oral mucosal disease.

## 3. Methods

### 3.1. Subject Recruitment and Sampling of Human Gingival Biopsies and Saliva

Human gingival biopsy samples were collected from study participants, including healthy uninfected controls (n = 3) and people living with HIV (PWH; n = 10), under a protocol approved by the Institutional Review Board of University Hospitals Cleveland Medical Center. Written informed consent was obtained from all participants prior to sample collection, as described previously [2,8,67]. Healthy control participants were 18 years of age or older and in good general health. Exclusion criteria included the presence of oral inflammatory conditions, such as gingivitis or periodontitis; a history or diagnosis of oral cancer; soft-tissue lesions; and tobacco use within the preceding month. Gingival tissue samples were processed to generate single-cell suspensions, which were subsequently prepared for single-cell RNA sequencing (scRNA-seq) according to the manufacturer’s protocols.

### 3.2. Single-Cell RNA Sequencing

Single-cell suspensions were fixed with 10x Genomics’ Fixed RNA Profiling fixation reagent. Libraries were prepared by the Applied Functional Genomics Core at Case Western Reserve University using 10x Genomics’ Chromium GEM-X Flex Gene Expression kits (human transcriptome probe set, 10x Genomics protocol CG000787), with one probe barcode assigned per donor and all samples pooled in a single reaction for a targeted recovery of 20,000 cells per donor. Library quality was assessed on an Agilent Fragment Analyzer. The pooled library was sequenced on an Illumina NovaSeq X 10B flow cell, paired-end with dual indexing, targeting a sequencing depth of 20,000 reads per cell.

### 3.3. Single-Cell Data Processing

Sequencing data were demultiplexed by probe barcode and aligned to the human reference genome with Cell Ranger (10x Genomics; version-9.0.1). Ambient RNA contamination was estimated and removed using FastCAR [68]. Quality control, normalization, integration, and UMAP dimension reduction were carried out in Seurat (version 5.2.0), as described previously [69]. Cell identities were assigned using SingleR with the Blueprint and ENCODE reference. Epithelial cells were selected from the annotated populations for the analyses shown here.

### 3.4. Pathway Analysis

Genes were ranked by their log fold change and signed log *p* value differential expression statistics for PWH versus Controls, and gene set enrichment analysis was performed with fgsea (https://bioconductor.org/packages/fgsea, accessed on 2 October 2026); against the WikiPathways [70] collection of ConsensusPathDB [71] and the Reactome [72] collection of the Molecular Signatures Database (MSigDB) [73]. Enriched pathways were selected by nominal *p* value. Normalized enrichment scores and gene-level false discovery rates are taken from the enrichment output, and leading edge genes are shown at their average log2 fold change between groups. Analyses and figures were generated in R.

## Data Availability

Single cell RNA sequencing data have been deposited in the Gene Expression Omnibus under accession [GSE349157], with raw sequence data in the Sequence Read Archive.
